# Supplementary material for: Verbal Autopsy with Participatory Action Research (VAPAR) programme in Mpumalanga, South Africa: protocol for evaluation
Source: BMJ Open. 2020 Feb 4;10(2):e036597. doi: 10.1136/bmjopen-2019-036597 (PMC7045152; doi:10.1136/bmjopen-2019-036597)
Supplement: Author's manuscript [file bmjopen-2019-036597.draft_revisions.pdf]

BMJ Open is committed to open peer review. As part of this commitment we make the peer review history of every article we publish publicly available.

When an article is published we post the peer reviewers' comments and the authors' responses online. We also post the versions of the paper that were used during peer review. These are the versions that the peer review comments apply to.

The versions of the paper that follow are the versions that were submitted during the peer review process. They are not the versions of record or the final published versions. They should not be cited or distributed as the published version of this manuscript.

BMJ Open is an open access journal and the full, final, typeset and author-corrected version of record of the manuscript is available on our site with no access controls, subscription charges or pay-per-view fees (http://bmjopen.bmj.com).

If you have any questions on BMJ Open's open peer review process please email info.bmjopen@bmj.com

BMJ Open

Verbal autopsy with participatory action research (VAPAR) programme in Mpumalanga, South Africa: protocol for evaluation

Journal:	*BMJ Open*
Manuscript ID	bmjopen-2019-036597
Article Type:	Protocol
Date Submitted by the Author:	20-Dec-2019

Complete List of Authors:	Witter, Sophie; Queen Margaret University, IGHD<br>Van Der Merwe, Maria; Independent consultant<br>Twine, Rhian; University of the Witwatersrand,<br>Mabetha, Denny; Wits University<br>Hove, Jennifer; Wits University<br>Goosen, Gerhard; Mpumalanga Department of Health<br>D'Ambruoso, L; University of Aberdeen, Centre for Global Development; Dr.

Keywords:	HEALTH SERVICES ADMINISTRATION & MANAGEMENT, Health policy < HEALTH SERVICES ADMINISTRATION & MANAGEMENT, International health services < HEALTH SERVICES ADMINISTRATION & MANAGEMENT, PUBLIC HEALTH, PRIMARY CARE, Organisation of health services < HEALTH SERVICES ADMINISTRATION & MANAGEMENT

SCHOLARONE™
Manuscripts

I, the Submitting Author has the right to grant and does grant on behalf of all authors of the Work (as defined in the below author licence), an exclusive licence and/or a non-exclusive licence for contributions from authors who are: i) UK Crown employees; ii) where BMJ has agreed a CC-BY licence shall apply, and/or iii) in accordance with the terms applicable for US Federal Government officers or employees acting as part of their official duties; on a worldwide, perpetual, irrevocable, royalty-free basis to BMJ Publishing Group Ltd ("BMJ") its licensees and where the relevant Journal is co-owned by BMJ to the co-owners of the Journal, to publish the Work in this journal and any other BMJ products and to exploit all rights, as set out in our licence.

The Submitting Author accepts and understands that any supply made under these terms is made by BMJ to the Submitting Author unless you are acting as an employee on behalf of your employer or a postgraduate student of an affiliated institution which is paying any applicable article publishing charge ("APC") for Open Access articles. Where the Submitting Author wishes to make the Work available on an Open Access basis (and intends to pay the relevant APC), the terms of reuse of such Open Access shall be governed by a Creative Commons licence – details of these licences and which Creative Commons licence will apply to this Work are set out in our licence referred to above.

Other than as permitted in any relevant BMJ Author's Self Archiving Policies, I confirm this Work has not been accepted for publication elsewhere, is not being considered for publication elsewhere and does not duplicate material already published. I confirm all authors consent to publication of this Work and authorise the granting of this licence.

For peer review only

Verbal autopsy with participatory action research (VAPAR) programme in Mpumalanga, South Africa: protocol for evaluation

Sophie Witter[1*], Maria van der Merwe[2], Rhian Twine[3], Denny Mabetha[3], Jennifer Hove[3], Gerhard Goosen[4], Lucia d'Ambruoso[5]

1. Institute for Global Health and Development, Queen Margaret University, Edinburgh, UK
2. Independent consultant
3. MRC/Wits Rural Public Health and Health Transitions Research Unit (Agincourt), School of Public Health, University of the Witwatersrand, South Africa
4. District Clinical Specialist Team, Ehlanzeni District, Mpumalanga Department of Health
5. Centre for Global Development and Institute of Applied Health Sciences, University of Aberdeen, Scotland, UK

* Corresponding author: IGHD, Queen Margaret University, Edinburgh, EH21 6UU, UK. Email: switter@qmu.ac.uk. Phone: +44 131474000; fax +44 1314740001.

Keywords: participatory action research; realist evaluation; verbal autopsy; health; South Africa

Authors' contributions: SW led on the development of the evaluation methods and drafting of the paper. LD, MvM, RT, JH, DM, GG worked on the development of the theory of change framework with SW. All authors contributed to the overall programme development, as well as the drafting and finalisation of the paper.

Funding: The research presented in this article is funded by the Health Systems Research Initiative from Department for International Development (DFID)/Medical Research Council (MRC)/Wellcome Trust/Economic and Social Research Council (ESRC), grant number MR/P014844/1.

Competing interests statement. The authors state that they have no competing interests.

Word Count: 3313

Abstract

Introduction

There is a growing recognition of the importance of developing learning health systems which can engage all stakeholders in cycles of evidence generation, reflection, action and learning from action to deal with adaptive problems. There is however limited evaluative evidence of approaches to developing or strengthening such systems, particularly in low- and middle-income settings. In this protocol, we aim to contribute to developing and sharing knowledge on models of building collaborative learning platforms through our evaluation of the VAPAR programme.

Methods and analysis

For peer review only

The evaluation takes a participatory approach, focussed on joint learning on whether and how VAPAR contributes to its aims, and what can be learned for this and similar settings. A realist-informed theory of change was developed by the research team as part of a broader collaboration with other stakeholders. The evaluation will draw on a wide variety of perspectives and data, including programme data and secondary data. This will be supplemented by in-depth interviews and workshops at the end of each cycle to probe the different domains, understand changes to the positions of different actors within the local health system, and feed back into improved learning and action in the next cycle. Quantitative data such as VA will be analysed for significant trends in health indicators for different population groups. However, the bulk of the data will be qualitative and will be analysed thematically.

Ethics and dissemination
Ethics in participatory approaches include a careful focus on the power relationships within the group, such that all groups are given voice and influence, in addition to the usual considerations of informed participation. Within the programme, we will focus on reflexivity, relationship building, two-way learning and learning from failure to reduce power imbalances and mitigate against a blame culture. Local engagement and change will be prioritised in dissemination.

Article summary
Strengths and limitations of study

- The evaluation will add to the limited literature on collaborative learning platforms to support learning health systems in low and middle income settings
- It will also build the field of evaluation of participatory research at multiple levels of the local health system
- The evaluation combines realist approaches with PAR and allows for testing and refinement over several cycles
- Risks related to power imbalances and insider/outsider tensions are acknowledged, with mitigating strategies planned
- Findings will be specific to this site but broader engagement is planned to allow for sharing of lessons with other learning sites using related models.

For peer review only

Introduction

Background on the VAPAR programme

The VAPAR programme started in 2017 in Mpumalanga Province, South Africa as a partnership of local and international researchers, community members and health system stakeholders. Its aim is to embed a system of knowledge production and exchange for health systems strengthening in order to improve services and outcomes for vulnerable group's health locally and with the potential, if successful, for wider learning, uptake and sustainability (www.vapar.org).

In VAPAR, data from Verbal Autopsy (VA) and Participatory Action Research (PAR) is combined in a series of reflection-and-action cycles based on continuous quality improvement for health systems strengthening, engaging relevant stakeholders at different levels of the health system (Figure 1). The programme consists of 3 learning-and-action cycles over 2017-22, with each cycle of the VAPAR programme including four stages: observe, analyse, plan and act (Hussain-Alkhateeb et al. 2019).

The VA component incorporates new World Health Organization (WHO) indicators developed with the Mpumalanga Department of Health during pilot work in 2015/16 on social and health system factors contributing to the 'circumstances of mortality' (Hussain-Alkhateeb et al. 2019) within VA data gathered through the MRC/Wits Agincourt Health and Socio-Demographic Surveillance System (HDSS) in Mpumalanga. These VA outputs are shared during PAR with village-based groups, which identify priority topics, analyse root causes and the impact the problem, identify stakeholders and plan action to address these with key stakeholder groups. During the pilot phase in 2016 of the programme, the areas of under-5 mortality and human immunodeficiency virus (HIV) infection were nominated by researchers and the Department of Health; however in the main phase communities nominated access to clean water, alongside alcohol and drug abuse as key local health priorities (Wariri et al. 2017; Hove et al. 2019; Brooks et al. 2017; Oladeinde et al. 2019). The last two topics were carried through by communities into the main phase in 2017. Data from VA and PAR have then been interpreted with district and provincial stakeholders (within the health sector but also beyond, as relevant) in order to reach common understanding on problems and root causes, leading to actionable agendas and promoting learning from that action in ongoing processes.

The research is informed by established post-positivist paradigms asserting that all truths are partial. The work is thus rooted in and draws from critical (Pawson and Tilley 1997), constructivist (Guba and Lincoln 2005), and participatory/cooperative traditions (Heron and Reason 1997). Through paradigmatic interweaving, we seek to deepen understanding in and of health systems as complex adaptive systems and social constructions (Paina and Peters 2012) located in and continually (re)shaped by wider social, political and historical contexts, and cooperative enquiry as political participation in collaborative practical knowing, action and transformation. Drawn together, the key underlying assumption is that practical, experiential knowledge that is co-constructed, self-reflective and embedded in complex, adaptive social and health systems will support and inform the organisation and delivery of public goods that are equity-oriented and people-centred. The beneficiaries are intended to

For peer review only

be people and practitioners in resource-constrained systems, collectively possessing rights and responsibilities for health, health care and wider public services.

Context

VAPAR is situated in Mpumalanga, South Africa, a rural province of 4.6 million people in the northeast, bordering Swaziland and Mozambique. Conditions in poor and rural villages are comparable with many other settings in the region: there is limited piped water, rudimentary sanitation, underdeveloped roads, unaffordable electricity and high unemployment (Kahn et al. 2012). The burden of HIV in South Africa is high and highly unequal. Prevalence in black populations is 40–50 times that of white and in adolescents, risks are 8 times higher in female adolescents than males (Shisana et al. 2014) . Age-adjusted HIV prevalence in the study area is 26% in women and 19% in men (Clark et al. 2015).

In spite of entrenched social and health inequities, the post-apartheid policy context in South Africa is progressive and inclusive. There is a constitutional commitment to the right to health and community participation for primary care (Department of Health. Republic of South Africa. 2004), and National Health Insurance (NHI) was launched in 2012 as a clear commitment to Universal Health Coverage (UHC) (Department of Health 2017). Despite a 'near-ideal' policy context, there is chronic underinvestment in public services. This has resulted in human resource crises, corruption, poor stewardship and deteriorating infrastructure – and deep disconnects between policy and implementation as a result (Coovadia et al. 2009). The health system also faces a complex 'quadruple' burden of socially patterned disease comprising: chronic infectious diseases (characterised by HIV/AIDS and tuberculosis), non-communicable conditions, maternal and child mortality, and mortality owing to injury and violence (Tollman et al. 2008; Statistics South Africa 2019).

Purposes of evaluation

In this paper, we lay out the approach which will be taken by our evaluation, which will be participatory among our key constituencies and will aim to understand whether and how VAPAR contributes to its aims, and what can be learned for this and similar settings. We present the theory of change of the programme, how it was developed and is evolving, and how it will be tracked using mixed methods. We discuss our positionality and some of the risks and ethical issues arising.

In addition to informing the development of the programme, the evaluation aims to build evidence on how to develop collaborative reflection and action for health through multiple levels of engagement and more authentic community engagement, which has been highlighted as a key gap area for health systems research (Erasmus et al. 2014) generally as well as in the province over the period of engagement. More broadly, it aims to enrich learning on PAR and its evaluation, as well as contributing to current debates on learning health systems (Witter and Jensen 2019) and on health system strengthening, with many of the areas of focus of VAPAR mapping onto suggested process goals for a stronger health system (Witter et al. 2019).

For peer review only

Methods and analysis

Evaluation approach

Participatory evaluation is a growing field (Cullen and Coryn 2011), however, evaluation of VAPAR faces additional challenges of evaluating an intervention which is itself participatory, multi-level, multi-cycle, pragmatic, emergent and embedded in rapidly changing contexts Our approach therefore includes the following elements, which reflect the programme design:

1. It is post-positivist in epistemology, recognising that knowledge is valid only relatively to a specific context, society, culture or individual and is socially (and potentially cooperatively) constructed (Guba and Lincoln 2005).
2. It is participatory and embedded in that it will build on reflections and insights of partners and wider stakeholders which are generated as part of the PAR cycles
3. It will be adaptive, to allow for changes in the programme and its environment which may occur over time
4. It is theory-based and looking for contribution[1] not attribution, starting from a hypothesised theory of change and examining actual changes against that
5. It draws from critical realist evaluation (Marchal et al. 2013; Pawson and Tilley 1997) in trying to identify mechanisms of change operating in specific contexts, and the outcomes to which they lead.
6. The focus on actors and institutions also allows us to probe into political economy factors – incentives, power relationships, ideas and ideologies (Harris and Booth 2013) – which will be important explanatory factors for why and how change does or does not occur.
7. We will in addition record resource intensity, including intangible costs for participants, as part of good practice for thinking about replication and scalability.

It is important to note that this is an evaluation of the VAPAR approach as a whole (a meta-evaluation, rather than focusing on local actions triggered individually). Methodological points of interest and innovation will include being able to test and refine our theory of change over repeated cycles, and being able to test the adaptation of realist approaches to participatory processes.

Stages of evaluation

Developing the initial theory of change

During the first PAR cycle in 2017-19, the research team developed an initial theory of change for VAPAR, based on continuous interactions with community, health system and wider public administration stakeholders over a number of prior years (in pre-pilot and pilot phases), as well as wider literature and secondary data. This theory of change considers the challenges and resources in the context, the expected causal pathways for addressing challenges, including change mechanisms and their underpinning assumptions, and desired

[1] (https://www.betterevaluation.org/en/plan/approach/contribution_analysis n.d.),

For peer review only - http://bmjopen.bmj.com/site/about/guidelines.xhtml

outcomes. The expected causal pathways were discussed with stakeholders at workshops in cycle 1 and start of cycle 2.

Testing and refining the theory

In each cycle, data will be collected which will allow us to refine our understanding of the intervention. This will include qualitative and quantitative data collected by the programme (on context, inputs, activities, outputs, outcomes and assumptions), supplemented by end-of-cycle interviews and workshops. These will be reviewed by the programme team and key stakeholders at the end of each cycle, leading to refined engagement and a more developed theory of change.

Final evaluation

This will bring together the learning from across the programme, describing the starting situation, the initial theory of change, how this was adapted over the years, the evidence of interplay of context, mechanisms, actors and outcomes, leading to the final theory of change for future testing, and to inform sustainability and potential scale up and replication.

Theory of change

Figure 2 presents the initial theory of change.

The context factors outline some of the key challenges which the programme is seeking to address – such as lack of constructive engagement between communities and health system and organisational culture issues within the health system – as well as some of the resources and opportunities, such as a progressive policy environment and a growing network of institutions collecting data on community health.

The inputs represent the envisaged contribution of the VAPAR programme in (1) supporting co-production of timely and relevant local evidence on health and other challenges faced by the communities in our focal area, and (2) enabling exchange and engagement with stakeholders within the health and public administration system to develop local solutions, collaboratively implement them and reflect on implementation.

In relation to mechanisms of change, three channels are hypothesised:

- Greater confidence in and commitment to co-producing and using evidence by all stakeholders, including as an input to services
- Improved relationships and trust between communities, researchers and health authorities
- Increased motivation and capacity for community involvement and localised evidence-based primary health care in health and other sectors

Outputs are expected to include the establishment of a learning platform or space, ownership and uptake of locally relevant evidence, and ensuing collective action and reflection on action in an ongoing manner. A learning platform in this context is understood

For peer review only

as a neutral, respectful forum in which to co-produce, exchange and use evidence for action, and to learn from that action.

Outcomes are broken into short-term changes, such as improved policy implementation within existing resources; medium-term changes, such as Improved health service organisation, resourcing and delivery; and long-term changes, such as improved health behaviours and outcomes, but also the sustaining and transfer of the learning from the programme.

Although these are presented in a linear fashion, it is clear that these stages are connected, fluid and in continuous interaction, with the mechanisms key to bringing about change. The learning cycles present opportunities to engage in and analyse these repeated interactions over time.

Underlying assumptions were identified, many of which are themselves potentially influenced by the programme, such as:

- the research institutions, Department of Health and local communities being able and willing to engage over time and being open to dialogue
- the three core constituencies having some flexibility of resources to be able to respond to new co-produced evidence
- there being sufficient social coherence to support movement towards shared priorities and actions
- there being sufficient stability in the health sector for receptivity to programme outputs
- there being a wider interest in distributed and collaborative PAR (in relation to transfer of lessons to other settings)

Evaluation methods

The main questions to be examined by the evaluation are summarised in Table 1 below, which also points to the data source for answering them.

The evaluation will be led by a team member who has some independence from the VAPAR process but who is embedded and able to facilitate reflections and learning from the main stakeholders in the research team, communities and health system. Different perspectives will be compared, noting synergies and tensions across the group. The focus will be on joint learning, and understanding the explanatory factors as much as the outputs and outcomes.

Most of the evaluation 'indicators' are qualitative, reflecting the focus of the programme on changing 'software' such as relationships, trust, attitudes and skills (for example, in communication and evidence use). Many will be extracted from routine programme and secondary data sources compiled by the research team throughout the VAPAR process.

For peer review only

Data analysis

Data collection and analysis will be continuous, collaborative and inclusive, with reviews at the end of each cycle to aid partner learning and inform improvements to VAPAR engagement (van der Merwe and et al 2020 still to be submitted). Results will be discussed with key actors from local to provincial level, such as community groups, PHC supervisors, facility managers, community health workers, front-line health staff, health programme and research managers and agencies in other sectors such as water and sanitation, housing and the environment.

Quantitative data such as VA will be analysed for significant trends in health indicators for different population groups. However, the bulk of the data will be qualitative and analysed thematically, focusing on understanding relevant changes, their drivers, their perceived impact on different groups and their relationships with one another, how they interact with other changing context features, any unintended consequences (including negative) and the implications for future interventions to build similar learning platforms.

Ethics and dissemination

While we emphasise a participatory approach, in line with our programme aims, this does create a risk of an overly optimistic assessment, with both insiders and external respondents seeking to emphasise positive outcomes. This risk will be mitigated by the continuity of data collection and the multitude of sources; findings on impact on collaborators will not, for example, be drawn from a single interview but from repeated observations and interactions across time. Regular practice of reflexive and self-critical thinking will also be practiced within the research team, drawing on our own learning regarding the construction of safe spaces in which constructive, respectful reflection and critique can be encouraged. A degree of insider/outsider tension (Olivier, Whyle, and Gilson 2018) is acknowledged during this process.

Risk may arise due to a lack of involvement or commitment on the part of participants in communities and the health systems, lack of data, and/or controversial and negative results. Steps will be taken to promote the evaluation through regular contact and dialogue with all involved. This will be underpinned by constructive accountability to mitigate against blame, negativity and a punitive focus, embracing failures as learning opportunities, and negative results as well as successes with a view to understanding key mechanisms in both.

Ethical approval has been obtained from the authors' institutes and from the provincial health authority. Principles of research ethics related to un-harmful ways to treat individuals will apply. Ethical considerations are also anticipated related to the interdisciplinary, relativist and transformative nature of the work, the less rigid distinctions between researchers, implementers and advocates, and the commitments to knowledge for action.

In each phase, there will be actions to minimise potential harm or negative consequences to participants. Informed consent will be gained from all participants. Participants will be informed about the nature of the research, its aims, objectives, procedures and outcomes. Participants will be assured that identifying information will be anonymised, and will not be disclosed beyond the research team without permission. Preliminary results will be fed back

For peer review only

to, and verified with, participants before being disseminated more widely. Participants will be reimbursed for time spent participating in the research via provision of subsistence and travel expenses. All participants will be free to leave the study at any time and for any reason. Efforts to develop partnerships and processes beyond the programme will be sought throughout.

Ethical arrangements that apply to the routine surveillance in MRC/Wits Agincourt Unit will apply to the VAs that will be acquired in the proposed research. Specific ethical issues relate to the PAR and health systems elements. PAR is underpinned by relativist and transformative epistemologies, and is a dynamic and context dependent process. These features may be unfamiliar to, or viewed as un-scientific by, medical research ethics committees and discussion may be necessary on these features, which may incur delays in the ethical approval process. Furthermore, PAR is concerned with transferring power through the research process towards those most directly affected by the issues investigated (Loewenson et al. 2014; Baum, MacDougall, and Smith 2006). Ethical conduct is therefore considered in terms of continual checking and rechecking of categories and dynamics of power by those intended to benefit from the process. It is also acknowledged that the changing of situations of social exclusion through empowerment gained via learning from knowledge and action may be open to stigmatisation and negative consequence in communities for individuals involved. Potential risks from participating in the long-term acting on information will be explicitly considered with participants, investigators and the International Steering Committee (which also supports quality assurance within the programme). We will work with the Agincourt Stakeholder Engagement Office in the event of disputes or other difficulties in transparent and constructive dialogue with communities or other stakeholders to address and resolve issues where necessary.

In the PAR and health systems consultations, protecting the identities of participants may not be possible or necessary. Time will be taken at the outset with participants to ensure that ethical principles are agreed, respected, implemented, revisiting fit and function during the process. Ethical challenges also arise related to anonymity and confidentiality of visual data. Participants using visual methods receive training on how and why to secure permissions from the subjects of images. Where photographic material is collected and used, separate release permissions will be secured.

The plans centralise the social nature of knowledge creation and transfer. Acknowledging that significant knowledge emerges from the combination of disparate perspectives (Rynes, Bartunek, and Daft 2001), time will be invested to build relationships, trust and shared understandings where partners accommodate and learn from different cultures and systems and where control is shifted as far as possible to stakeholder groups. It is acknowledged that operating between diverse sectors and perspectives may introduce problems. Where inter-sectoral tensions are identified, they will be supportively and constructively addressed. The process will invest in understanding and aligning perspectives, acknowledging and accommodating differences and distinguishing roles, with the overall purpose of identifying and progressing collective agendas through partnerships that span boundaries for positive change. Potential difficulties will be mitigated against through a supportive and well-structured process, protected time, reliable data, shared dialogue, effective training and dedicated staff underpinned by principles of two-way learning (Rynes,

For peer review only

Bartunek, and Daft 2001). If conflicts, tensions or problems ultimately threaten the process, ad-hoc sessions (in person wherever possible) will be held. The focus will be to respect and safeguard the partnerships. If the process fails, then reasons why and lessons learned will be documented as a contribution to the methodological literature (D'Ambruoso et al. 2017).

Acknowledging the relevance of where and how outputs are disseminated, reporting will be balanced between academic practitioner literature and public media. Partner voices will be given space wherever possible, seeking to privilege the 'local gaze' (Hullur et al. 2016) and provide lessons which support further local action and benefits. Quantitative data on VA will be available for scrutiny through Agincourt DSS, however qualitative data will be curated by the programme team. Findings will be shared through local meetings, briefs, social media sites, conferences and academic publications. Wider collaboration and lesson sharing with other centres in South Africa and beyond which are testing learning health system models is also planned.

References

Baum, Fran, Colin MacDougall, and Danielle Smith. 2006. "Participatory Action Research." *Journal of Epidemiology and Community Health* 60 (10): 854–57. https://doi.org/10.1136/jech.2004.028662.

Brooks, Chloe, Lucia D'Ambruoso, Karolina Kazimierczak, Sizzy Ngobeni, Rhian Twine, Stephen Tollman, Kathleen Kahn, and Peter Byass. 2017. "Introducing Visual Participatory Methods to Develop Local Knowledge on HIV in Rural South Africa." *BMJ Global Health* 2 (3): e000231. https://doi.org/10.1136/bmjgh-2016-000231.

Clark, Samuel J., F. Xavier Gómez-Olivé, Brian Houle, Margaret Thorogood, Kerstin Klipstein-Grobusch, Nicole Angotti, Chodziwadziwa Kabudula, Jill Williams, Jane Menken, and Stephen Tollman. 2015. "Cardiometabolic Disease Risk and HIV Status in Rural South Africa: Establishing a Baseline." *BMC Public Health* 15 (1): 1–9. https://doi.org/10.1186/s12889-015-1467-1.

Coovadia, Hoosen, Rachel Jewkes, Peter Barron, David Sanders, and Diane McIntyre. 2009. "The Health and Health System of South Africa: Historical Roots of Current Public Health Challenges." *The Lancet* 374 (9692): 817–34. https://doi.org/10.1016/S0140-6736(09)60951-X.

Cullen, Anne, and Chris L. S. Coryn. 2011. "Forms and Functions of Participatory Evaluation in International Development: A Review of the Empirical and Theoretical Literature No Title." *Journal of MultiDisciplinary Evaluation* 7 (16): 32–47.

D'Ambruoso, Lucia, Ties Boerma, Peter Byass, Edward Fottrell, Kobus Herbst, Karin Källander, and Zoë Mullan. 2017. "The Case for Verbal Autopsy in Health Systems Strengthening." *The Lancet Global Health* 5 (1): e20–21. https://doi.org/10.1016/S2214-109X(16)30332-1.

Department of Health. Republic of South Africa. 2004. *No. 61 of 2003: National Health Act, 2004.* https://www.up.ac.za/media/shared/12/ZP_Files/health-act.zp122778.pdf.

Department of Health, Republic of South Africa. 2017. *National Health Insurance for South Africa. Towards Universal Health Coverage.* http://www.health.gov.za/index.php/nhi-documents?download=2257:white-paper-nhi-2017.

Erasmus, E., M. Orgill, H. Schneider, and L. Gilson. 2014. "Mapping the Existing Body of Health Policy Implementation Research in Lower Income Settings: What Is Covered and What Are the Gaps?" *Health Policy and Planning* 29 (suppl 3): iii35–50. https://doi.org/10.1093/heapol/czu063.

For peer review only

Guba, Egon G., and Yvonna S. Lincoln. 2005. "Paradigmatic Controversies, Contradictions, and Emerging Confluences." In *N. K. Denzin & Y. S. Lincoln (Eds.), The Sage Handbook of Qualitative Research*, 191–215. SAGE Publications Ltd.

Harris, Daniel, and David Booth. 2013. "Methods and Resources Applied Political Economy Analysis: Five Practical Issues."

Heron, J, and P Reason. 1997. "A Participatory Inquiry Paradigm." *Qualitative Inquiry* 3 (3): 274–94.

Hove, J, L D'Ambruoso, D Mabetha, and ... 2019. "'Water Is Life': Developing Community Participation for Clean Water in Rural South Africa." *BMJ Global ...*, 1–13. https://doi.org/10.1136/bmjgh-2018-001377.

https://www.betterevaluation.org/en/plan/approach/contribution_analysis. n.d. "Contribution Analysis | Better Evaluation." Accessed November 24, 2019. https://www.betterevaluation.org/en/plan/approach/contribution_analysis.

Hullur, Nitya, Lucia D'Ambruoso, Kerstin Edin, Ryan G Wagner, Sizzy Ngobeni, Kathleen Kahn, Stephen Tollman, and Peter Byass. 2016. "Community Perspectives on HIV, Violence and Health Surveillance in Rural South Africa: A Participatory Pilot Study." *Journal of Global Health* 6 (1). https://doi.org/10.7189/jogh.06.010406.

Hussain-Alkhateeb, Laith, Lucia D Ambruoso, Stephen Tollman, Maria Van Der Merwe, Rhian Twine, Linus Schiöler, Max Petzold, et al. 2019. "Enhancing the Value of Mortality Data for Health Systems : Adding Circumstances Of Mortality CATegories (COMCATs) to Deaths Investigated by Verbal Autopsy Enhancing the Value of Mortality Data for Health Systems : Adding Verbal Autopsy." *Global Health Action* 12 (1). https://doi.org/10.1080/16549716.2019.1680068.

Kahn, Kathleen, Mark A. Collinson, F. Xavier Gómez-olivé, Obed Mokoena, Rhian Twine, Paul Mee, Sulaimon A. Afolabi, et al. 2012. "Profile: Agincourt Health and Socio-Demographic Surveillance System." *International Journal of Epidemiology* 41 (4): 988–1001. https://doi.org/10.1093/ije/dys115.

Loewenson, R, A Laurell, C Hogstedt, L D'Ambruoso, and Z Shroff. 2014. *Participatory Action Research in Health Systems A Methods Reader*. TARSC, AHPSR, WHO, IDRC Canada, EQUINET, Harare.

Marchal, Bruno, Sara Van Belle, Vincent De Brouwere, and Sophie Witter. 2013. "Studying Complex Interventions: Reflections from the FEMHealth Project on Evaluating Fee Exemption Policies in West Africa and Morocco." https://doi.org/10.1186/1472-6963-13-469.

Merwe, M van der, L D'Ambruoso, S Witter, R Twine, D Mabetha, J Hove, D Mdluli, et al. 2020. "Evaluation of a Collaborative Learning Platform to Strengthen Rural Primary Health Care: A Review of the VAPAR Programme in Mpumalanga, South Africa." *Health Systems & Reform (Submitting Early 2020)*.

Oladeinde, O, D Mabetha, R Twine, J Hove, M van der Merwe, P Byass, S Witter, K Kahn, and L D'Ambruoso. 2019. "Progressing Participatory Action Research to Reduce Alcohol and Other Drug Abuse in Rural South Africa: Reflections on an Action and Learning Process No Title." *Global Health Action (under Review)*.

Olivier, J, E Whyle, and L Gilson. 2018. "Embedded Health Policy and Systems Research: A Rapid Scoping Review. Report for the Alliance for Health Policy and Systems Research." *Alliance for Health Policy and System Research* October: 1–20.

Paina, L., and D. H. Peters. 2012. "Understanding Pathways for Scaling up Health Services through the Lens of Complex Adaptive Systems." *Health Policy and Planning* 27 (5): 365–73. https://doi.org/10.1093/heapol/czr054.

Pawson, R., and N. Tilley. 1997. *Realistic Evaluation.* London: Sage Publications.

Rynes, S.L, J.M Bartunek, and R.L. Daft. 2001. "Across the Great Divide: Knowledge

For peer review only

Creation and Transfer between Practitioners and Academics." *Academy of Management Journal* 44: 340–55.

Shisana, O., T Rehle, Simbayi L.C., K. Zuma, S. Jooste, N Zungu, D Labadarios, and D Onoya. 2014. "South African National HIV Prevalence, Incidence and Behaviour Survey, 2012." Cape Town. http://www.hsrc.ac.za/en/research-outputs/view/6871.

Statistics South Africa. 2019. "Mortality and Causes of Death in South Africa, 2016: Findings from Death Notification."

Tollman, Stephen M., Kathleen Kahn, Benn Sartorius, Mark A. Collinson, Samuel J. Clark, and Michel L. Garenne. 2008. "Implications of Mortality Transition for Primary Health Care in Rural South Africa: A Population-Based Surveillance Study." *The Lancet* 372 (9642): 893–901. https://doi.org/10.1016/S0140-6736(08)61399-9.

Wariri, O, L D'Ambruoso, R Twine, S Ngobeni, M van der Merwe, B Spies, K Kahn, S Tollman, R G Wagner, and P Byass. 2017. "Initiating a Participatory Action Research Process in the Agincourt Health and Socio–Demographic Surveillance Site." *Journal of Global Health* 7 (1). https://doi.org/10.7189/jogh.07.010413.

Witter, S., and C. Jensen. 2019. "Learning Health Systems in Low and Middle Income Countries: Background Paper for Alliance for Health Policy and Systems Research, Geneva." Geneva.

Witter, S. et al. (2019) Health system strengthening – reflections on its meaning, assessment and our state of knowledge. *International Journal of Health Planning and Management*, 1-10

For peer review only

Figure 1 VAPAR action learning cycle

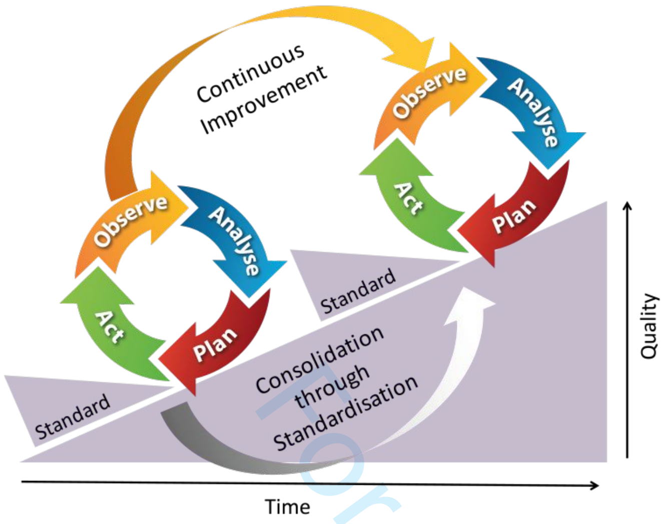

For peer review only

Figure 2 Initial theory of change, VAPAR

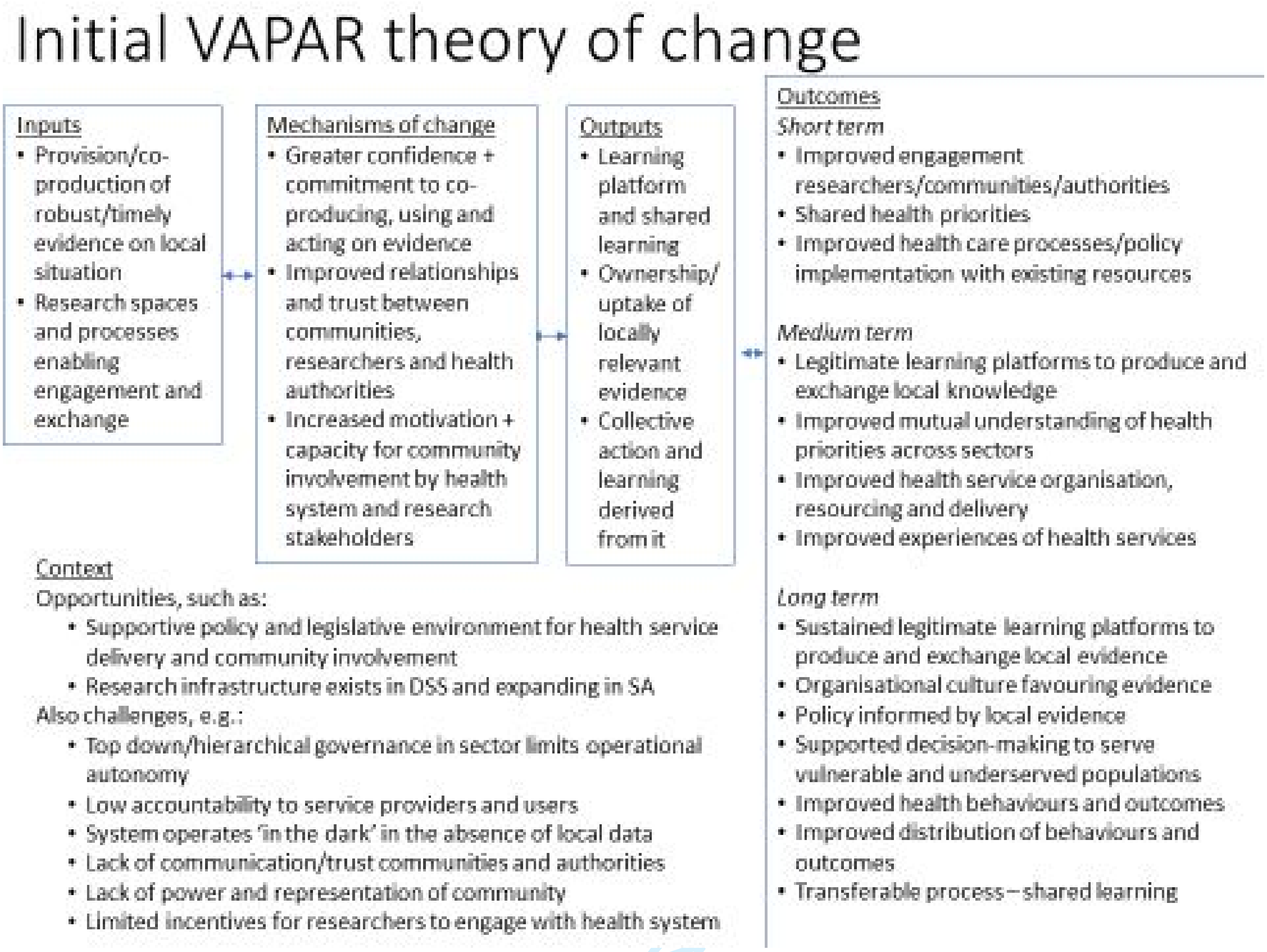

Table 1 Evaluation questions

Domain	Evaluation focus/indicators	Sources
Inputs		
• Provision/co-production of robust/timely evidence on local situation<br>• Research spaces and processes enabling engagement and exchange of local (and wider, as relevant) evidence	• Degree and quality of stakeholder engagement (by whom, and for which activities; participant understanding and perception of processes)<br>• Relevance of data which is co-produced for local needs (match to known burdens of disease, link to priority and actionable topics etc.)<br>• Intensity of activities and match to programme plans (number of community meetings, meetings with health system stakeholders etc.)<br>• Use of budgeted resources by all partners<br>• Changes made to programme approach based on learning through activities	• Research briefs<br>• Reflection from communities and authorities during each stage<br>• Summary programme reports<br>• End of each cycle review interviews/workshops<br>• Published VAPAR research papers<br>• Social media platforms – The Conversation pieces etc.<br>• Programme budget and expenditure
Mechanisms		
• Greater confidence/ commitment to co-producing, using and acting on evidence by all stakeholders<br>• Improved relationships and trust between communities, researchers and health authorities<br>• Increased motivation/capacity for community involvement by health system and research stakeholders	• Jointly authored outputs<br>• Regularity of meetings and other collaboration between stakeholders<br>• Changes to stakeholder perceptions of relevance of partnership and evidence<br>• Changes to stakeholder skills, engagement, confidence and self-efficacy, self-reported and as observed during interactions<br>• Changes to stakeholder relationships (e.g. better communication, less hierarchical blockages and punitive relationships)	• Participant feedback<br>• End of cycle interviews/workshops<br>• Systematic noting of observations and reflections on change from team members<br>• Important emails stored in shared drive<br>• Invitations to events between partners<br>• Stakeholder mapping by team and study participants, repeated over time to record change

For peer review only

	• Any process changes noted (including for wider Agincourt DSS – e.g. more proactive engagement with health system actors)	
Outputs		
• Learning platform<br>• Ownership/uptake of locally relevant evidence<br>• Collective action and learning derived from it	• Continued commitment to process (e.g. attendance and active participation at meetings and in joint activities – in claimed and invited spaces)<br>• Behaviour change by any of key stakeholders (greater focus on uptake by researchers, greater use of evidence by system, more community inputs into both)<br>• Collective action plans and extent of their completion	• Programme reports<br>• Local action plans and follow up reporting<br>• End of cycle interviews/workshops
Outcomes		
Short term/foundational<br>• Improved engagement researchers/communities/authorities<br>• Improved awareness of and shared local health priorities<br>• Improved health care processes/policy implementation with existing resources	• Evidence of demand for continued exchange by all stakeholders (e.g. independent meetings or collaboration, not linked to VAPAR)<br>• Value given to different forms of evidence and inclusion of different evidence in decision making processes<br>• More evidence citation and use in local planning and review within health and other relevant sectors<br>• Inclusive strategic review and reflection used to plan and prioritise locally<br>• Changes to service planning and organisation linked to VAPAR and VAPAR-inspired processes	• Programme reports and team observations<br>• Cycle 3 interviews/workshops<br>• Secondary reports, e.g. District Health Plan, Annual Performance Plan, Integrated Development Plans, quarterly and annual reviews<br>• Local Action Plans and other PAR outputs (e.g. Photovoice, root causes mapping, Venn diagrams)<br>• Engagement and collaboration by other research in MRC/Wits-Agincourt

For peer review only

Medium term<br>• Legitimate learning platforms to produce and exchange local knowledge<br>• Improved understanding of and commitment to equitable health priorities, including across sectors<br>• Improved health service organisation, resourcing and delivery<br>• Improved understanding of and experiences of health services by users	• Perceptions of stakeholders on ownership, utility, impact on them personally of VAPAR-catalysed exchanges<br>• Any notable changes in resources mobilised for health and how these are used<br>• Reductions in reported problems for health services in study area (e.g. stock-outs, unfilled posts, but also for outreach activities, e.g. increased effectiveness of CHWs)<br>• User satisfaction increased, as expressed through PAR, client surveys, reduction in community protests etc.	• Cycle 3 interviews/workshops<br>• Quarterly district health review reports on challenges, changes in organisation, resource use for each year<br>• Satisfaction trends (from routine reports, VAPAR data, any relevant additional Agincourt data, press reports)
Long term<br>• Sustained legitimate learning platforms to produce and exchange local evidence<br>• Organisational culture favouring evidence of different types (within health system and research institutions)<br>• Supported decision-making to serve vulnerable and underserved populations<br>• Policy and planning informed by local evidence<br>• Improved health behaviours and outcomes<br>• Improved distribution of behaviours and outcomes<br>• Transferable process – shared learning	• Continued support for VAPAR-inspired fora and activities<br>• Greater use of local data in policy and programme documents in province<br>• Clearer focus on marginalised communities in provincial health plans and reporting<br>• Engaging and relationship building across sectors, horizontally and vertically, in support of Primary Health Care (PHC)<br>• Greater access to and utilisation of essential health services<br>• Reduced morbidity and mortality<br>• Any other social impacts raised by participants<br>• Trends in inequity: health outcomes, behaviours and services will be assessed in terms of gender, age, ethnicity and income	• PAR narratives and visual data<br>• Other relevant research reports (including VAPAR publications tracking specific health issues and training materials)<br>• Funds to sustain and progress VA, PAR, local health policy and systems research, VAPAR<br>• Provincial and district health plans and reports<br>• District Health Information System (DHIS) data disaggregated<br>• HDSS data disaggregated<br>• VA data - trends<br>• South African Population Research Infrastructure Network (SAPRIN) health care utilisation data

For peer review only

	• Dissemination and training materials and activities, deployed nationally and internationally<br>• Uptake of VAPAR learning and approach in other provinces of South Africa (e.g. through SAPRIN, the new network of HDSS sites in South Africa, or other health system research centres) and potentially beyond	• Reports by programme partners (e.g. WHO, StatsSA, INDEPTH network, Code4SouthArica)
Context		
Relevant changes to context will be tracked, including:<br>Opportunities, such as:<br>• Supportive policy and legislative environment for health service delivery and community involvement<br>• Research infrastructure exists in HDSS and expanding in South Africa<br>Also challenges, e.g.:<br>• Top down/hierarchical governance in sector limits operational autonomy<br>• Low accountability to service providers and users<br>• System operates 'in the dark' in the absence of local data<br>• Lack of communication/trust communities and authorities<br>• Lack of power and representation of community<br>• Limited incentives for researchers to engage with health system		• Policy documents, including national (e.g. on NHI, PHC Re-engineering, and relating to relevant other sectors, such as water and alcohol and drugs)<br>• Annual provincial health expenditure data (from annual reports)<br>• End of cycle interviews/workshops<br>• Programme documents and data<br>• Wider literature<br>• VAPAR outputs, e.g. tracking of decision space in the province for health)<br>• Social media and other interactions such as webinars<br>• Research infrastructure development – e.g. SAPRIN/Agincourt

For peer review only

	• Systematic noting of observations and reflections on change by team members<br>• News articles<br>• Other MRC/Wits Agincourt Unit research

For peer review only

BMJ Open

Verbal autopsy with participatory action research (VAPAR) programme in Mpumalanga, South Africa: protocol for evaluation

Journal:	*BMJ Open*
Manuscript ID	bmjopen-2019-036597.R1
Article Type:	Protocol
Date Submitted by the Author:	14-Jan-2020
Complete List of Authors:	Witter, Sophie; Queen Margaret University, IGHD<br>Van Der Merwe, Maria; Independent consultant<br>Twine, Rhian; University of the Witwatersrand,<br>Mabetha, Denny; Wits University<br>Hove, Jennifer; Wits University<br>Goosen, Gerhard; Mpumalanga Department of Health<br>D’Ambruoso, L; University of Aberdeen, Centre for Global Development; Dr.
**Primary Subject Heading**:	Health services research
Secondary Subject Heading:	Global health, Health policy, Public health, Qualitative research
Keywords:	HEALTH SERVICES ADMINISTRATION & MANAGEMENT, Health policy < HEALTH SERVICES ADMINISTRATION & MANAGEMENT, International health services < HEALTH SERVICES ADMINISTRATION & MANAGEMENT, PUBLIC HEALTH, PRIMARY CARE, Organisation of health services < HEALTH SERVICES ADMINISTRATION & MANAGEMENT

SCHOLARONE™
Manuscripts

I, the Submitting Author has the right to grant and does grant on behalf of all authors of the Work (as defined in the below author licence), an exclusive licence and/or a non-exclusive licence for contributions from authors who are: i) UK Crown employees; ii) where BMJ has agreed a CC-BY licence shall apply, and/or iii) in accordance with the terms applicable for US Federal Government officers or employees acting as part of their official duties; on a worldwide, perpetual, irrevocable, royalty-free basis to BMJ Publishing Group Ltd ("BMJ") its licensees and where the relevant Journal is co-owned by BMJ to the co-owners of the Journal, to publish the Work in this journal and any other BMJ products and to exploit all rights, as set out in our licence.

The Submitting Author accepts and understands that any supply made under these terms is made by BMJ to the Submitting Author unless you are acting as an employee on behalf of your employer or a postgraduate student of an affiliated institution which is paying any applicable article publishing charge ("APC") for Open Access articles. Where the Submitting Author wishes to make the Work available on an Open Access basis (and intends to pay the relevant APC), the terms of reuse of such Open Access shall be governed by a Creative Commons licence – details of these licences and which Creative Commons licence will apply to this Work are set out in our licence referred to above.

Other than as permitted in any relevant BMJ Author's Self Archiving Policies, I confirm this Work has not been accepted for publication elsewhere, is not being considered for publication elsewhere and does not duplicate material already published. I confirm all authors consent to publication of this Work and authorise the granting of this licence.

For peer review only

Verbal autopsy with participatory action research (VAPAR) programme in Mpumalanga, South Africa: protocol for evaluation

Sophie Witter[1*], Maria van der Merwe[2], Rhian Twine[3], Denny Mabetha[3], Jennifer Hove[3], Gerhard Goosen[4], Lucia d'Ambruoso[5]

1. Institute for Global Health and Development, Queen Margaret University, Edinburgh, UK
2. Independent consultant
3. MRC/Wits Rural Public Health and Health Transitions Research Unit (Agincourt), School of Public Health, University of the Witwatersrand, South Africa
4. District Clinical Specialist Team, Ehlanzeni District, Mpumalanga Department of Health
5. Centre for Global Development and Institute of Applied Health Sciences, University of Aberdeen, Scotland, UK

* Corresponding author: IGHD, Queen Margaret University, Edinburgh, EH21 6UU, UK. Email: switter@qmu.ac.uk. Phone: +44 131474000; fax +44 1314740001.

Keywords: participatory action research; realist evaluation; verbal autopsy; health; South Africa

Authors' contributions: SW led on the development of the evaluation methods and drafting of the paper. LD, MvM, RT, JH, DM, GG worked on the development of the theory of change framework with SW. All authors contributed to the overall programme development, as well as the drafting and finalisation of the paper.

Funding: The research presented in this article is funded by the Health Systems Research Initiative from Department for International Development (DFID)/Medical Research Council (MRC)/Wellcome Trust/Economic and Social Research Council (ESRC), grant number MR/P014844/1.

Competing interests statement. The authors state that they have no competing interests.

Word Count: 3313

Abstract

Introduction

There is a growing recognition of the importance of developing learning health systems which can engage all stakeholders in cycles of evidence generation, reflection, action and learning from action to deal with adaptive problems. There is however limited evaluative evidence of approaches to developing or strengthening such systems, particularly in low- and middle-income settings. In this protocol, we aim to contribute to developing and sharing knowledge on models of building collaborative learning platforms through our evaluation of the VAPAR programme.

Methods and analysis

For peer review only

For peer review only - http://bmjopen.bmj.com/site/about/guidelines.xhtml

The evaluation takes a participatory approach, focussed on joint learning on whether and how VAPAR contributes to its aims, and what can be learned for this and similar settings. A realist-informed theory of change was developed by the research team as part of a broader collaboration with other stakeholders. The evaluation will draw on a wide variety of perspectives and data, including programme data and secondary data. This will be supplemented by in-depth interviews and workshops at the end of each cycle to probe the different domains, understand changes to the positions of different actors within the local health system, and feed back into improved learning and action in the next cycle. Quantitative data such as VA will be analysed for significant trends in health indicators for different population groups. However, the bulk of the data will be qualitative and will be analysed thematically.

Ethics and dissemination
Ethics in participatory approaches include a careful focus on the power relationships within the group, such that all groups are given voice and influence, in addition to the usual considerations of informed participation. Within the programme, we will focus on reflexivity, relationship building, two-way learning and learning from failure to reduce power imbalances and mitigate against a blame culture. Local engagement and change will be prioritised in dissemination.

Strengths and limitations of study

- It combines realist approaches with PAR and allows for testing and refinement over several cycles
- Risks related to power imbalances and insider/outsider tensions are acknowledged, with mitigating strategies planned
- It aims to add to the limited literature on collaborative learning platforms to support learning health systems in low and middle income settings
- The study will also build the field of evaluation of participatory research at multiple levels of the local health system
- One limitation is that results will be specific to this site, however broader engagement is planned to allow for sharing of lessons with other learning sites using related models

For peer review only

Introduction

Background on the VAPAR programme

The VAPAR programme started in 2017 in Mpumalanga Province, South Africa as a partnership of local and international researchers, community members and health system stakeholders. Its aim is to embed a system of knowledge production and exchange for health systems strengthening in order to improve services and outcomes for vulnerable group's health locally and with the potential, if successful, for wider learning, uptake and sustainability (www.vapar.org).

In VAPAR, data from Verbal Autopsy (VA) and Participatory Action Research (PAR) is combined in a series of reflection-and-action cycles based on continuous quality improvement for health systems strengthening, engaging relevant stakeholders at different levels of the health system (Figure 1). The programme consists of 3 learning-and-action cycles over 2017-22, with each cycle of the VAPAR programme including four stages: observe, analyse, plan and act (1).

The VA component incorporates new World Health Organization (WHO) indicators developed with the Mpumalanga Department of Health during pilot work in 2015/16 on social and health system factors contributing to the 'circumstances of mortality' (1) within VA data gathered through the MRC/Wits Agincourt Health and Socio-Demographic Surveillance System (HDSS) in Mpumalanga. These VA outputs are shared during PAR with village-based groups, which identify priority topics, analyse root causes and the impact the problem, identify stakeholders and plan action to address these with key stakeholder groups. During the pilot phase in 2016 of the programme, the areas of under-5 mortality and human immunodeficiency virus (HIV) infection were nominated by researchers and the Department of Health; however in the main phase communities nominated access to clean water, alongside alcohol and drug abuse as key local health priorities (2–4). The last two topics were carried through by communities into the main phase in 2017. Data from VA and PAR have then been interpreted with district and provincial stakeholders (within the health sector but also beyond, as relevant) in order to reach common understanding on problems and root causes, leading to actionable agendas and promoting learning from that action in ongoing processes.

The research is informed by established post-positivist paradigms asserting that all truths are partial. The work is thus rooted in and draws from critical (5), constructivist (6), and participatory/cooperative traditions (7). Through paradigmatic interweaving, we seek to deepen understanding in and of health systems as complex adaptive systems and social constructions (8) located in and continually (re)shaped by wider social, political and historical contexts, and cooperative enquiry as political participation in collaborative practical knowing, action and transformation. Drawn together, the key underlying assumption is that practical, experiential knowledge that is co-constructed, self-reflective and embedded in complex, adaptive social and health systems will support and inform the organisation and delivery of public goods that are equity-oriented and people-centred. The beneficiaries are intended to be people and practitioners in resource-constrained systems, collectively possessing rights and responsibilities for health, health care and wider public services.

For peer review only

Context

VAPAR is situated in Mpumalanga, South Africa, a rural province of 4.6 million people in the northeast, bordering Swaziland and Mozambique. Conditions in poor and rural villages are comparable with many other settings in the region: there is limited piped water, rudimentary sanitation, underdeveloped roads, unaffordable electricity and high unemployment (9). The burden of HIV in South Africa is high and highly unequal. Prevalence in black populations is 40–50 times that of white and in adolescents, risks are 8 times higher in female adolescents than males (10) . Age-adjusted HIV prevalence in the study area is 26% in women and 19% in men (11).

In spite of entrenched social and health inequities, the post-apartheid policy context in South Africa is progressive and inclusive. There is a constitutional commitment to the right to health and community participation for primary care (12), and National Health Insurance (NHI) was launched in 2012 as a clear commitment to Universal Health Coverage (UHC) (13). Despite a 'near-ideal' policy context, there is chronic underinvestment in public services. This has resulted in human resource crises, corruption, poor stewardship and deteriorating infrastructure – and deep disconnects between policy and implementation as a result (14). The health system also faces a complex 'quadruple' burden of socially patterned disease comprising: chronic infectious diseases (characterised by HIV/AIDS and tuberculosis), non-communicable conditions, maternal and child mortality, and mortality owing to injury and violence (15,16).

Purposes of evaluation

In this paper, we lay out the approach which will be taken by our evaluation, which will be participatory among our key constituencies and will aim to understand whether and how VAPAR contributes to its aims, and what can be learned for this and similar settings. We present the theory of change of the programme, how it was developed and is evolving, and how it will be tracked using mixed methods. We discuss our positionality and some of the risks and ethical issues arising.

In addition to informing the development of the programme, the evaluation aims to build evidence on how to develop collaborative reflection and action for health through multiple levels of engagement and more authentic community engagement, which has been highlighted as a key gap area for health systems research (17) generally as well as in the province over the period of engagement. More broadly, it aims to enrich learning on PAR and its evaluation, as well as contributing to current debates on learning health systems (18) and on health system strengthening, with many of the areas of focus of VAPAR mapping onto suggested process goals for a stronger health system (18).

Methods and analysis

Evaluation approach

Participatory evaluation is a growing field (19), however, evaluation of VAPAR faces additional challenges of evaluating an intervention which is itself participatory, multi-level,

For peer review only

multi-cycle, pragmatic, emergent and embedded in rapidly changing contexts Our approach therefore includes the following elements, which reflect the programme design:

1. It is post-positivist in epistemology, recognising that knowledge is valid only relatively to a specific context, society, culture or individual and is socially (and potentially cooperatively) constructed (6).
2. It is participatory and embedded in that it will build on reflections and insights of partners and wider stakeholders which are generated as part of the PAR cycles
3. It will be adaptive, to allow for changes in the programme and its environment which may occur over time
4. It is theory-based and looking for contribution[1] not attribution, starting from a hypothesised theory of change and examining actual changes against that
5. It draws from critical realist evaluation (5,20) in trying to identify mechanisms of change operating in specific contexts, and the outcomes to which they lead.
6. The focus on actors and institutions also allows us to probe into political economy factors – incentives, power relationships, ideas and ideologies (21) – which will be important explanatory factors for why and how change does or does not occur.
7. We will in addition record resource intensity, including intangible costs for participants, as part of good practice for thinking about replication and scalability.

It is important to note that this is an evaluation of the VAPAR approach as a whole (a meta-evaluation, rather than focusing on local actions triggered individually). Methodological points of interest and innovation will include being able to test and refine our theory of change over repeated cycles, and being able to test the adaptation of realist approaches to participatory processes.

Stages of evaluation

Developing the initial theory of change

During the first PAR cycle in 2017-19, the research team developed an initial theory of change for VAPAR, based on continuous interactions with community, health system and wider public administration stakeholders over a number of prior years (in pre-pilot and pilot phases), as well as wider literature and secondary data. This theory of change considers the challenges and resources in the context, the expected causal pathways for addressing challenges, including change mechanisms and their underpinning assumptions, and desired outcomes. The expected causal pathways were discussed with stakeholders at workshops in cycle 1 and start of cycle 2.

Testing and refining the theory

In each cycle, data will be collected which will allow us to refine our understanding of the intervention. This will include qualitative and quantitative data collected by the programme

[1] https://www.betterevaluation.org/en/plan/approach/contribution_analysis n.d

(on context, inputs, activities, outputs, outcomes and assumptions), supplemented by end-of-cycle interviews and workshops. These will be reviewed by the programme team and key stakeholders at the end of each cycle, leading to refined engagement and a more developed theory of change.

Final evaluation

This will bring together the learning from across the programme, describing the starting situation, the initial theory of change, how this was adapted over the years, the evidence of interplay of context, mechanisms, actors and outcomes, leading to the final theory of change for future testing, and to inform sustainability and potential scale up and replication.

Theory of change

Figure 2 presents the initial theory of change.

The context factors outline some of the key challenges which the programme is seeking to address – such as lack of constructive engagement between communities and health system and organisational culture issues within the health system – as well as some of the resources and opportunities, such as a progressive policy environment and a growing network of institutions collecting data on community health.

The inputs represent the envisaged contribution of the VAPAR programme in (1) supporting co-production of timely and relevant local evidence on health and other challenges faced by the communities in our focal area, and (2) enabling exchange and engagement with stakeholders within the health and public administration system to develop local solutions, collaboratively implement them and reflect on implementation.

In relation to mechanisms of change, three channels are hypothesised:

- Greater confidence in and commitment to co-producing and using evidence by all stakeholders, including as an input to services
- Improved relationships and trust between communities, researchers and health authorities
- Increased motivation and capacity for community involvement and localised evidence-based primary health care in health and other sectors

Outputs are expected to include the establishment of a learning platform or space, ownership and uptake of locally relevant evidence, and ensuing collective action and reflection on action in an ongoing manner. A learning platform in this context is understood as a neutral, respectful forum in which to co-produce, exchange and use evidence for action, and to learn from that action.

Outcomes are broken into short-term changes, such as improved policy implementation within existing resources; medium-term changes, such as Improved health service organisation, resourcing and delivery; and long-term changes, such as improved health behaviours and outcomes, but also the sustaining and transfer of the learning from the programme.

For peer review only

Although these are presented in a linear fashion, it is clear that these stages are connected, fluid and in continuous interaction, with the mechanisms key to bringing about change. The learning cycles present opportunities to engage in and analyse these repeated interactions over time.

Underlying assumptions were identified, many of which are themselves potentially influenced by the programme, such as:

- the research institutions, Department of Health and local communities being able and willing to engage over time and being open to dialogue
- the three core constituencies having some flexibility of resources to be able to respond to new co-produced evidence
- there being sufficient social coherence to support movement towards shared priorities and actions
- there being sufficient stability in the health sector for receptivity to programme outputs
- there being a wider interest in distributed and collaborative PAR (in relation to transfer of lessons to other settings)

Evaluation methods

The main questions to be examined by the evaluation are summarised in Table 1 below, which also points to the data source for answering them.

The evaluation will be led by a team member who has some independence from the VAPAR process but who is embedded and able to facilitate reflections and learning from the main stakeholders in the research team, communities and health system. Different perspectives will be compared, noting synergies and tensions across the group. The focus will be on joint learning, and understanding the explanatory factors as much as the outputs and outcomes.

Most of the evaluation 'indicators' are qualitative, reflecting the focus of the programme on changing 'software' such as relationships, trust, attitudes and skills (for example, in communication and evidence use). Many will be extracted from routine programme and secondary data sources compiled by the research team throughout the VAPAR process.

Data analysis

Data collection and analysis will be continuous, collaborative and inclusive, with reviews at the end of each cycle to aid partner learning and inform improvements to VAPAR engagement. Results will be discussed with key actors from local to provincial level, such as community groups, PHC supervisors, facility managers, community health workers, front-line health staff, health programme and research managers and agencies in other sectors such as water and sanitation, housing and the environment.

Quantitative data such as VA will be analysed for significant trends in health indicators for different population groups. However, the bulk of the data will be qualitative and analysed

For peer review only

thematically, focusing on understanding relevant changes, their drivers, their perceived impact on different groups and their relationships with one another, how they interact with other changing context features, any unintended consequences (including negative) and the implications for future interventions to build similar learning platforms.

Patient and public involvement

The VAPAR programme emerged from and further develops participatory action research pilots working with community members in the district, so members of the public (not patients in this context) were integrally involved in its shaping, in the selection of priority topics, in the generation of evidence and its interpretation, as well as participating in discussion and dissemination events. This approach will continue to be followed in the evaluation activities, as it is a core component of our programme and evaluation approach.

Ethics and dissemination

While we emphasise a participatory approach, in line with our programme aims, this does create a risk of an overly optimistic assessment, with both insiders and external respondents seeking to emphasise positive outcomes. This risk will be mitigated by the continuity of data collection and the multitude of sources; findings on impact on collaborators will not, for example, be drawn from a single interview but from repeated observations and interactions across time. Regular practice of reflexive and self-critical thinking will also be practiced within the research team, drawing on our own learning regarding the construction of safe spaces in which constructive, respectful reflection and critique can be encouraged. A degree of insider/outsider tension (22) is acknowledged during this process.

Risk may arise due to a lack of involvement or commitment on the part of participants in communities and the health systems, lack of data, and/or controversial and negative results. Steps will be taken to promote the evaluation through regular contact and dialogue with all involved. This will be underpinned by constructive accountability to mitigate against blame, negativity and a punitive focus, embracing failures as learning opportunities, and negative results as well as successes with a view to understanding key mechanisms in both.

Ethical approval has been obtained from the authors' institutes and from the provincial health authority. Principles of research ethics related to un-harmful ways to treat individuals will apply. Ethical considerations are also anticipated related to the interdisciplinary, relativist and transformative nature of the work, the less rigid distinctions between researchers, implementers and advocates, and the commitments to knowledge for action.

In each phase, there will be actions to minimise potential harm or negative consequences to participants. Informed consent will be gained from all participants. Participants will be informed about the nature of the research, its aims, objectives, procedures and outcomes. Participants will be assured that identifying information will be anonymised, and will not be disclosed beyond the research team without permission. Preliminary results will be fed back to, and verified with, participants before being disseminated more widely. Participants will be reimbursed for time spent participating in the research via provision of subsistence and travel expenses. All participants will be free to leave the study at any time and for any

For peer review only

reason. Efforts to develop partnerships and processes beyond the programme will be sought throughout.

Ethical arrangements that apply to the routine surveillance in MRC/Wits Agincourt Unit will apply to the VAs that will be acquired in the proposed research. Specific ethical issues relate to the PAR and health systems elements. PAR is underpinned by relativist and transformative epistemologies, and is a dynamic and context dependent process. These features may be unfamiliar to, or viewed as un-scientific by, medical research ethics committees and discussion may be necessary on these features, which may incur delays in the ethical approval process. Furthermore, PAR is concerned with transferring power through the research process towards those most directly affected by the issues investigated (23,24). Ethical conduct is therefore considered in terms of continual checking and rechecking of categories and dynamics of power by those intended to benefit from the process. It is also acknowledged that the changing of situations of social exclusion through empowerment gained via learning from knowledge and action may be open to stigmatisation and negative consequence in communities for individuals involved. Potential risks from participating in the long-term acting on information will be explicitly considered with participants, investigators and the International Steering Committee (which also supports quality assurance within the programme). We will work with the Agincourt Stakeholder Engagement Office in the event of disputes or other difficulties in transparent and constructive dialogue with communities or other stakeholders to address and resolve issues where necessary.

In the PAR and health systems consultations, protecting the identities of participants may not be possible or necessary. Time will be taken at the outset with participants to ensure that ethical principles are agreed, respected, implemented, revisiting fit and function during the process. Ethical challenges also arise related to anonymity and confidentiality of visual data. Participants using visual methods receive training on how and why to secure permissions from the subjects of images. Where photographic material is collected and used, separate release permissions will be secured.

The plans centralise the social nature of knowledge creation and transfer. Acknowledging that significant knowledge emerges from the combination of disparate perspectives (25), time will be invested to build relationships, trust and shared understandings where partners accommodate and learn from different cultures and systems and where control is shifted as far as possible to stakeholder groups. It is acknowledged that operating between diverse sectors and perspectives may introduce problems. Where inter-sectoral tensions are identified, they will be supportively and constructively addressed. The process will invest in understanding and aligning perspectives, acknowledging and accommodating differences and distinguishing roles, with the overall purpose of identifying and progressing collective agendas through partnerships that span boundaries for positive change. Potential difficulties will be mitigated against through a supportive and well-structured process, protected time, reliable data, shared dialogue, effective training and dedicated staff underpinned by principles of two-way learning (25). If conflicts, tensions or problems ultimately threaten the process, ad-hoc sessions (in person wherever possible) will be held. The focus will be to respect and safeguard the partnerships. If the process fails, then

For peer review only

reasons why and lessons learned will be documented as a contribution to the methodological literature (26).

Acknowledging the relevance of where and how outputs are disseminated, reporting will be balanced between academic practitioner literature and public media. Partner voices will be given space wherever possible, seeking to privilege the ‘local gaze’ (27) and provide lessons which support further local action and benefits. Quantitative data on VA will be available for scrutiny through Agincourt DSS, however qualitative data will be curated by the programme team. Findings will be shared through local meetings, briefs, social media sites, conferences and academic publications. Wider collaboration and lesson sharing with other centres in South Africa and beyond which are testing learning health system models is also planned.

For peer review only

References

1. Hussain-Alkhateeb L, Ambruoso LD, Tollman S, Merwe M Van Der, Twine R, Schiöler L, et al. Enhancing the value of mortality data for health systems : adding Circumstances Of Mortality CATegories (COMCATs) to deaths investigated by verbal autopsy Enhancing the value of mortality data for health systems : adding verbal autopsy. Glob Health Action [Internet]. 2019;12(1). Available from: https://doi.org/10.1080/16549716.2019.1680068
2. Wariri O, D’Ambruoso L, Twine R, Ngobeni S, van der Merwe M, Spies B, et al. Initiating a participatory action research process in the Agincourt health and socio–demographic surveillance site. J Glob Health [Internet]. 2017;7(1). Available from: http://jogh.org/documents/issue201701/jogh-07-010413.pdf
3. Hove J, D’Ambruoso L, Mabetha D, ... “Water is life”: developing community participation for clean water in rural South Africa. BMJ Glob [Internet]. 2019;1–13. Available from: https://abdn.pure.elsevier.com/en/publications/water-is-life-developing-community-participation-for-clean-water-
4. Brooks C, D’Ambruoso L, Kazimierczak K, Ngobeni S, Twine R, Tollman S, et al. Introducing visual participatory methods to develop local knowledge on HIV in rural South Africa. BMJ Glob Heal [Internet]. 2017;2(3):e000231. Available from: http://gh.bmj.com/lookup/doi/10.1136/bmjgh-2016-000231
5. Pawson R, Tilley N. Realistic Evaluation. London: Sage Publications; 1997.
6. Guba EG, Lincoln YS. Paradigmatic Controversies, Contradictions, and Emerging Confluences. In: N K Denzin & Y S Lincoln (Eds), The Sage handbook of qualitative research. SAGE Publications Ltd; 2005. p. 191–215.
7. Heron J, Reason P. A participatory inquiry paradigm. Qual Inq. 1997;3(3):274–94.
8. Paina L, Peters DH. Understanding pathways for scaling up health services through the lens of complex adaptive systems. Health Policy Plan [Internet]. 2012 Aug 1 [cited 2019 Nov 22];27(5):365–73. Available from: https://academic.oup.com/heapol/article-lookup/doi/10.1093/heapol/czr054
9. Kahn K, Collinson MA, Xavier Gómez-olivé F, Mokoena O, Twine R, Mee P, et al. Profile: Agincourt health and socio-demographic surveillance system. Int J Epidemiol. 2012;41(4):988–1001.
10. Shisana O, Rehle T, Simbayi L.C., Zuma K, Jooste S, Zungu N, et al. South African National HIV Prevalence, Incidence and Behaviour Survey, 2012. [Internet]. Cape Town; 2014 [cited 2019 Nov 24]. Available from: http://www.hsrc.ac.za/en/research-outputs/view/6871
11. Clark SJ, Gómez-Olivé FX, Houle B, Thorogood M, Klipstein-Grobusch K, Angotti

N, et al. Cardiometabolic disease risk and HIV status in rural South Africa: Establishing a baseline. BMC Public Health. 2015;15(1):1–9.
12. Department of Health. Republic of South Africa. No. 61 of 2003: National Health Act, 2004. [Internet]. 2004. Available from: https://www.up.ac.za/media/shared/12/ZP_Files/health-act.zp122778.pdf
13. Department of Health R of SA. National Health Insurance for South Africa. Towards universal health coverage. [Internet]. 2017. Available from: http://www.health.gov.za/index.php/nhi-documents?download=2257:white-paper-nhi-2017
14. Coovadia H, Jewkes R, Barron P, Sanders D, McIntyre D. The health and health system of South Africa: historical roots of current public health challenges. Lancet. 2009;374(9692):817–34.
15. Tollman SM, Kahn K, Sartorius B, Collinson MA, Clark SJ, Garenne ML. Implications of mortality transition for primary health care in rural South Africa: a population-based surveillance study. Lancet. 2008;372(9642):893–901.
16. Statistics South Africa. Mortality and causes of death in South Africa, 2016: Findings from death notification. 2019.
17. Erasmus E, Orgill M, Schneider H, Gilson L. Mapping the existing body of health policy implementation research in lower income settings: what is covered and what are the gaps? Health Policy Plan [Internet]. 2014 Dec 1 [cited 2019 Nov 24];29(suppl 3):iii35–50. Available from: https://academic.oup.com/heapol/article-lookup/doi/10.1093/heapol/czu063
18. Witter S, Jensen C. Learning health systems in low and middle income countries: background paper for Alliance for Health Policy and Systems Research, Geneva. Geneva; 2019.
19. Cullen A, Coryn CLS. Forms and Functions of Participatory Evaluation in International Development: A Review of the Empirical and Theoretical Literature No Title. J Multidiscip Eval. 2011;7(16):32–47.
20. Marchal B, Van Belle S, De Brouwere V, Witter S. Studying complex interventions: reflections from the FEMHealth project on evaluating fee exemption policies in West Africa and Morocco [Internet]. 2013 [cited 2019 Nov 24]. Available from: http://www.biomedcentral.com/1472-6963/13/469
21. Harris D, Booth D. Methods and Resources Applied political economy analysis: five practical issues. 2013.
22. Olivier J, Whyle E, Gilson L. Embedded Health Policy and Systems Research: A Rapid Scoping Review. Report for the Alliance for Health Policy and Systems Research. Alliance Heal Policy Syst Res. 2018;October:1–20.
23. Loewenson R, Laurell A, Hogstedt C, D'Ambruoso L, Shroff Z. Participatory action research in health systems A methods reader. TARSC, AHPSR, WHO, IDRC Canada, EQUINET, Harare; 2014.
24. Baum F, MacDougall C, Smith D. Participatory action research. J Epidemiol Community Health. 2006;60(10):854–7.
25. Rynes S., Bartunek J., Daft RL. Across the Great Divide: knowledge creation and transfer between practitioners and academics. Acad Manag J. 2001;44:340–55.
26. D'Ambruoso L, Boerma T, Byass P, Fottrell E, Herbst K, Källander K, et al. The case for verbal autopsy in health systems strengthening. Lancet Glob Heal. 2017;5(1):e20–1.
27. Hullur N, D'Ambruoso L, Edin K, Wagner RG, Ngobeni S, Kahn K, et al. Community perspectives on HIV, violence and health surveillance in rural South Africa: a participatory pilot study. J Glob Health [Internet]. 2016;6(1). Available from:

For peer review only

http://www.jogh.org/documents/issue201601/jogh-06-010406.pdf

For peer review only

Table 1 Evaluation questions

Domain	Evaluation focus/indicators	Sources
Inputs		
• Provision/co-production of robust/timely evidence on local situation<br>• Research spaces and processes enabling engagement and exchange of local (and wider, as relevant) evidence	• Degree and quality of stakeholder engagement (by whom, and for which activities; participant understanding and perception of processes)<br>• Relevance of data which is co-produced for local needs (match to known burdens of disease, link to priority and actionable topics etc.)<br>• Intensity of activities and match to programme plans (number of community meetings, meetings with health system stakeholders etc.)<br>• Use of budgeted resources by all partners<br>• Changes made to programme approach based on learning	• Research briefs<br>• Reflection from communities and authorities during each stage<br>• Summary programme reports<br>• End of each cycle review interviews/workshops<br>• Published VAPAR research papers<br>• Social media platforms – The Conversation pieces etc.<br>• Programme budget and expenditure

For peer review only

	through activities	
Mechanisms		
• Greater confidence/ commitment to co-producing, using and acting on evidence by all stakeholders<br>• Improved relationships and trust between communities, researchers and health authorities<br>• Increased motivation/capacity for community involvement by health system and research stakeholders	• Jointly authored outputs<br>• Regularity of meetings and other collaboration between stakeholders<br>• Changes to stakeholder perceptions of relevance of partnership and evidence<br>• Changes to stakeholder skills, engagement, confidence and self-efficacy, self-reported and as observed during interactions<br>• Changes to stakeholder relationships (e.g. better communication, less hierarchical blockages and punitive relationships)<br>• Any process changes noted (including for wider Agincourt DSS – e.g. more proactive	• Participant feedback<br>• End of cycle interviews/workshops<br>• Systematic noting of observations and reflections on change from team members<br>• Important emails stored in shared drive<br>• Invitations to events between partners<br>• Stakeholder mapping by team and study participants, repeated over time to record change

For peer review only

	engagement with health system actors)	
Outputs		
• Learning platform<br>• Ownership/uptake of locally relevant evidence<br>• Collective action and learning derived from it	• Continued commitment to process (e.g. attendance and active participation at meetings and in joint activities – in claimed and invited spaces)<br>• Behaviour change by any of key stakeholders (greater focus on uptake by researchers, greater use of evidence by system, more community inputs into both)<br>• Collective action plans and extent of their completion	• Programme reports<br>• Local action plans and follow up reporting<br>• End of cycle interviews/workshop s
Outcomes		
Short term/foundational<br>• Improved engagement researchers/communities/authoritie s<br>• Improved awareness of and shared local health priorities<br>• Improved health care processes/policy implementation with existing resources	• Evidence of demand for continued exchange by all stakeholders (e.g. independent meetings or collaboration, not linked to VAPAR)<br>• Value given to different forms	• Programme reports and team observations<br>• Cycle 3 interviews/workshop s<br>• Secondary reports, e.g. District Health Plan, Annual Performance Plan, Integrated Development Plans,

For peer review only

	of evidence and inclusion of different evidence in decision making processes<br>• More evidence citation and use in local planning and review within health and other relevant sectors<br>• Inclusive strategic review and reflection used to plan and prioritise locally<br>• Changes to service planning and organisation linked to VAPAR and VAPAR-inspired processes	quarterly and annual reviews<br>• Local Action Plans and other PAR outputs (e.g. Photovoice, root causes mapping, Venn diagrams)<br>• Engagement and collaboration by other research in MRC/Wits-Agincourt
Medium term<br>• Legitimate learning platforms to produce and exchange local knowledge<br>• Improved understanding of and commitment to equitable health priorities, including across sectors<br>• Improved health service organisation, resourcing and delivery<br>• Improved understanding of and experiences of health services by users	• Perceptions of stakeholders on ownership, utility, impact on them personally of VAPAR-catalysed exchanges<br>• Any notable changes in resources mobilised for health and how these are used<br>• Reductions in reported problems for health services	• Cycle 3 interviews/workshop s<br>• Quarterly district health review reports on challenges, changes in organisation, resource use for each year<br>• Satisfaction trends (from routine reports, VAPAR data, any relevant additional Agincourt data, press reports)

For peer review only

	in study area (e.g. stock-outs, unfilled posts, but also for outreach activities, e.g. increased effectiveness of CHWs)<br>• User satisfaction increased, as expressed through PAR, client surveys, reduction in community protests etc.	
Long term<br>• Sustained legitimate learning platforms to produce and exchange local evidence<br>• Organisational culture favouring evidence of different types (within health system and research institutions)<br>• Supported decision-making to serve vulnerable and underserved populations<br>• Policy and planning informed by local evidence<br>• Improved health behaviours and outcomes<br>• Improved distribution of behaviours and outcomes<br>• Transferable process – shared learning	• Continued support for VAPAR-inspired fora and activities<br>• Greater use of local data in policy and programme documents in province<br>• Clearer focus on marginalised communities in provincial health plans and reporting<br>• Engaging and relationship building across sectors, horizontally and vertically, in support of Primary Health Care (PHC)<br>• Greater access to and	• PAR narratives and visual data<br>• Other relevant research reports (including VAPAR publications tracking specific health issues and training materials)<br>• Funds to sustain and progress VA, PAR, local health policy and systems research, VAPAR<br>• Provincial and district health plans and reports<br>• District Health Information System (DHIS) data disaggregated<br>• HDSS data disaggregated<br>• VA data - trends<br>• South African Population Research Infrastructure Network (SAPRIN)

For peer review only

	utilisation of essential health services<br>• Reduced morbidity and mortality<br>• Any other social impacts raised by participants<br>• Trends in inequity: health outcomes, behaviours and services will be assessed in terms of gender, age, ethnicity and income<br>• Dissemination and training materials and activities, deployed nationally and internationally<br>• Uptake of VAPAR learning and approach in other provinces of South Africa (e.g. through SAPRIN, the new network of HDSS sites in South Africa, or other health system research centres) and potentially beyond	health care utilisation data<br>• Reports by programme partners (e.g. WHO, StatsSA, INDEPTH network, Code4SouthArica)
Context		

For peer review only

Relevant changes to context will be tracked, including:<br>Opportunities, such as:<br>• Supportive policy and legislative environment for health service delivery and community involvement<br>• Research infrastructure exists in HDSS and expanding in South Africa<br>Also challenges, e.g.:<br>• Top down/hierarchical governance in sector limits operational autonomy<br>• Low accountability to service providers and users<br>• System operates 'in the dark' in the absence of local data<br>• Lack of communication/trust communities and authorities<br>• Lack of power and representation of community<br>• Limited incentives for researchers to engage with health system	• Policy documents, including national (e.g. on NHI, PHC Re-engineering, and relating to relevant other sectors, such as water and alcohol and drugs)<br>• Annual provincial health expenditure data (from annual reports)<br>• End of cycle interviews/workshops<br>• Programme documents and data<br>• Wider literature<br>• VAPAR outputs, e.g. tracking of decision space in the province for health)<br>• Social media and other interactions such as webinars<br>• Research infrastructure development – e.g. SAPRIN/Agincourt<br>• Systematic noting of observations and reflections on change by team members<br>• News articles<br>• Other MRC/Wits Agincourt Unit research

Captions

Figure 1 VAPAR action learning cycle
Figure 2 Initial VAPAR theory of change

For peer review only

Figure 1 VAPAR action learning cycle

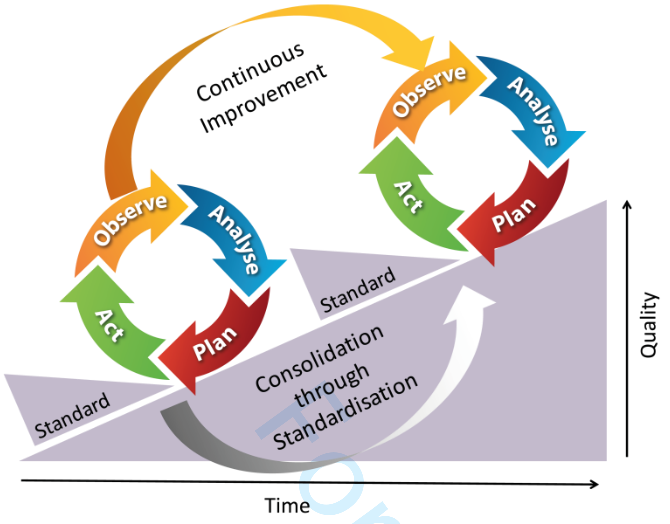

For peer review only

Initial VAPAR theory of change

Inputs

- Provision/co-production of robust/timely evidence on local situation
- Research spaces and processes enabling engagement and exchange

Mechanisms of change

- Greater confidence + commitment to co-producing, using and acting on evidence
- Improved relationships and trust between communities, researchers and health authorities
- Increased motivation + capacity for community involvement by health system and research stakeholders

Outputs

- Learning platform and shared learning
- Ownership/ uptake of locally relevant evidence
- Collective action and learning derived from it

Outcomes

Short term

- Improved engagement researchers/communities/authorities
- Shared health priorities
- Improved health care processes/policy implementation with existing resources

Medium term

- Legitimate learning platforms to produce and exchange local knowledge
- Improved mutual understanding of health priorities across sectors
- Improved health service organisation, resourcing and delivery
- Improved experiences of health services

Long term

- Sustained legitimate learning platforms to produce and exchange local evidence
- Organisational culture favouring evidence
- Policy informed by local evidence
- Supported decision-making to serve vulnerable and underserved populations
- Improved health behaviours and outcomes
- Improved distribution of behaviours and outcomes
- Transferable process – shared learning

Context

Opportunities, such as:

- Supportive policy and legislative environment for health service delivery and community involvement
- Research infrastructure exists in DSS and expanding in SA

Also challenges, e.g.:

- Top down/hierarchical governance in sector limits operational autonomy
- Low accountability to service providers and users
- System operates 'in the dark' in the absence of local data
- Lack of communication/trust communities and authorities
- Lack of power and representation of community
- Limited incentives for researchers to engage with health system
