# Supplementary material for: Verbal Autopsy with Participatory Action Research (VAPAR) programme in Mpumalanga, South Africa: protocol for evaluation
Source: BMJ Open. 2020 Feb 4;10(2):e036597. doi: 10.1136/bmjopen-2019-036597 (PMC7045152; doi:10.1136/bmjopen-2019-036597)
Supplement: Reviewer comments [file bmjopen-2019-036597.reviewer_comments.pdf]

PEER REVIEW HISTORY

BMJ Open publishes all reviews undertaken for accepted manuscripts. Reviewers are asked to complete a checklist review form (**http://bmjopen.bmj.com/site/about/resources/checklist.pdf**) and are provided with free text boxes to elaborate on their assessment. These free text comments are reproduced below.

ARTICLE DETAILS

**TITLE (PROVISIONAL)**	Verbal autopsy with participatory action research (VAPAR) programme in Mpumalanga, South Africa: protocol for evaluation
**AUTHORS**	Witter, Sophie; Van Der Merwe, Maria; Twine, Rhian; Mabetha, Denny; Hove, Jennifer; Goosen, Gerhard; D'Ambruoso, L

This article was not externally reviewed at BMJ Open. Protocols that have been independently assessed prior to submission to BMJ Open are usually fast-tracked to publication on the grounds that further substantial changes will not be possible. This independent assessment will usually be external, independent review for both a substantial grant award from a non-commercial or government funder and ethics approval.
